# Poster Session I - A45 0HOST RESPONSE TO BACTERIA INDUCES A SHIFT TOWARDS THE ENTEROENDOCRINE CELL LINEAGE IN MURINE ENTEROIDS

**DOI:** 10.1093/jcag/gwaf042.045

**Published:** 2026-02-13

**Authors:** M Nissan, S Girardin

**Affiliations:** University of Toronto Temerty Faculty of Medicine, Toronto, ON, Canada; University of Toronto Temerty Faculty of Medicine, Toronto, ON, Canada

## Abstract

**Background:**

*Shigella flexneri* is a leading cause of bacterial dysentery, disproportionately affecting low-resource settings. While its interaction with host cells has been extensively studied in immortalized cell lines, responses in primary intestinal epithelial systems remain poorly understood. Mice deficient in the innate immune sensor NLRC4 are highly susceptible to *S. flexneri*, offering a physiologically relevant model to examine host-pathogen dynamics in the gut.

**Aims:**

To investigate how primary intestinal epithelial cells respond to *S. flexneri* infection and to determine the role of NLRC4 in shaping these responses, particularly in the context of bacterial sensing, inflammation, and epithelial cell lineage dynamics.

**Methods:**

Ileal organoids were derived from wild-type (WT) and NLRC4-deficient (*Nlrc4*^-/-^) C57 BL/6 mice. Organoids were exposed to either invasive or non-invasive strains of *S. flexneri*, including bacterial supernatants. Intracellular bacterial growth, epithelial uptake, and transcriptional responses were assessed, including lineage-specific gene expression analysis.

**Results:**

NLRC4 was necessary to restrict intracellular growth of *S. flexneri* in organoids. Unexpectedly, WT organoids also responded to cell-free *S. flexneri* supernatants, suggesting NLRC4-dependent detection of type III secretion system components even in the absence of bacterial invasion. In *Nlrc4*^-/-^ organoids, both invasive and non-invasive *S. flexneri* were internalized, indicating a potential for intestinal epithelial cell-mediated bacterial uptake. Transcriptomic analysis revealed suppression of inflammatory pathways in infected *Nlrc4*_-/-_ organoids, coupled with increased expression of enteroendocrine cell markers. Similar transcriptional shifts were observed upon exposure to a non-pathogenic *S. flexneri* strain, but not bacterial supernatants, indicating that enteroendocrine cell expansion may be driven by bacterial contact or uptake.

**Conclusions:**

Primary murine intestinal epithelial cells exhibit distinct mechanisms of pathogen detection and response, including NLRC4-mediated sensing of bacterial effectors prior to invasion. The expansion of enteroendocrine cells during infection suggests a novel role for this cell lineage in host-pathogen interactions. These findings highlight the importance of studying gut immunity in physiologically relevant epithelial systems to better understand host responses to commensal and pathogenic microbes.

**Funding Agencies:**

CCC, CIHR

